# Physical activity of UK adults with chronic disease: cross-sectional analysis of accelerometer-measured physical activity in 96 706 UK Biobank participants

**DOI:** 10.1093/ije/dyy294

**Published:** 2019-02-05

**Authors:** Joseph Barker, Karl Smith Byrne, Aiden Doherty, Charlie Foster, Kazem Rahimi, Rema Ramakrishnan, Mark Woodward, Terence Dwyer

**Affiliations:** 1 The George Institute for Global Health, University of Oxford, Oxford, UK; 2 International Agency for Research on Cancer, Genetic Epidemiology Group, Lyon, France; 3 Big Data Institute, Li Ka Shing Centre for Health Information and Discovery, Nuffield Department of Population Health, University of Oxford, Oxford, UK; 4 Centre for Exercise, Nutrition and Health Sciences, University of Bristol, Bristol, UK; 5 The George Institute for Global Health, UNSW Sydney, Sydney, NSW, Australia

**Keywords:** Physical activity, accelerometry, chronic disease

## Abstract

**Background:**

Physical inactivity is associated with an increased risk of major chronic diseases, although uncertainty exists about which chronic diseases, themselves, might contribute to physical inactivity. The objective of this study was to compare the physical activity of those with chronic diseases to healthy individuals using an objective measure of physical activity.

**Methods:**

We conducted a cross-sectional analysis of data from 96 706 participants aged 40 years or older from the UK Biobank prospective cohort study (2006–10). Diagnoses were identified through ICD 9 and 10 coding within hospital admission records and a cancer registry linked to UK Biobank participants. We extracted summary physical activity information from participants who wore a wrist-worn triaxial accelerometer for 7 days. Statistical analyses included computation of adjusted geometric means and means using general linear models.

**Results:**

Participants with chronic disease undertook 9% or 61 minutes (95% confidence interval: 57.8–64.8) less moderate activity and 11% or 3 minutes (95% confidence interval: 2.7–3.3) less vigorous activity per week than individuals without chronic disease. Participants in every chronic-disease subgroup undertook less physical activity than those without chronic disease. Sixty-seven diagnoses within these subgroups were associated with lower moderate activity.

**Conclusions:**

The cross-sectional association of physical activity with chronic disease is broad. Given the substantial health benefits of being physically active, clinicians and policymakers should be aware that their patients with any chronic disease are at greater health risk from other diseases than anticipated because of their physical inactivity.

**Key Messages**

- Almost all patients with a chronic disease undertook less physical activity than healthy individuals.
- Participants with any chronic disease engaged in 6% less moderate and 11% less vigorous activity/week (geometric mean for chronic disease vs without) than individuals without chronic disease.
- Individuals with mental health disorders had the lowest moderate activity (559 minutes/week) compared with individuals without chronic disease (705.1 minutes/week).
- Individuals with cardiovascular diseases had the lowest vigorous activity (23.1 minutes/week) compared with individuals without chronic disease (27.0 minutes/week).

## Introduction

Physical inactivity is globally recognized as an independent risk factor for major chronic diseases.^1^ The Healthy People 2020 goals and the UK NICE guidelines specifically refer to the importance of increasing physical activity (PA) as part of the management of several chronic diseases such as cardiovascular disease, diabetes and musculoskeletal disorders.^2^^,^^3^ However, the evidence base on which assumptions about inactivity in specific disease subgroups are founded is limited and what is available has been based on subjective questionnaire reports that suffer from substantial random and non-random measurement error.^4^ The reference standard for measuring free-living energy expenditure, and thus PA, is doubly labelled water (DLW). However, DLW measurements are expensive and complex.^5^ Objective PA assessment with accelerometers is a good low-cost alternative to DLW and captures validated information on habitual levels of physical activity.^6^

We aimed to compare the PA of those with various chronic diseases to healthy individuals using accelerometer data from the UK Biobank study. The UK Biobank has collected the world’s largest objective PA dataset to date using wrist-worn accelerometers on a cohort of 103 720 participants with links to various health records in the UK.^7^^,^^8^ This database, therefore, provides a unique opportunity to reliably compare any potential difference in PA between participants with both common and rarer chronic disease against their healthy peers.

## Methods

### Study design and participants

The UK Biobank data set includes data from 502 656 UK adults between 40 and 69 years of age at recruitment (2006–10) via a centrally coordinated invitation from population-based National Health Service patient registers.^8^^,^^9^ Data were obtained by application to the UK Biobank, reference number 15 856. UK Biobank obtained ethical approval from the North West Multicentre Research Ethics Committee, the National Information Governance Board for Health and Social Care in England and Wales, and the Community Health Index Advisory Group in Scotland. All participants provided written informed consent.

### Exposures

Prevalence of chronic disease was identified using International Classification of Diseases, Ninth and Tenth Revisions (ICD 9 and 10) primary and secondary diagnoses from hospital admission data in England, Scotland and Wales and cancer registry data from 1997 up to accelerometer wear time for each individual (2013–15). The ICD codes were organized into 12 chronic-disease subgroups by ICD Chapters. These included neoplasms; infectious and parasitic diseases; diseases of the circulatory system; mental and behavioural disorders; diseases of the eye and adnexa; diseases of the ear and mastoid process; diseases of the blood and blood-forming organs and certain disorders involving the immune mechanism; diseases of the digestive system; diseases of the genitourinary system; diseases of the musculoskeletal system and connective tissue, endocrine, nutritional and metabolic diseases; diseases of the nervous system; and diseases of the respiratory system.^10^^,^^11^ Specific ICD codes were taken from these chapters in combination to identify specific disorders; see Supplementary materials section IC*D codes*, available as Supplementary data at *IJE* online. We did not select patients on the basis that they have one and only one disease out of all possible diseases. To enable us to capture the main associations of PA with a particular chronic disease, a person was defined as having a specific disease if they suffered from that disease and that disease only within the chronic-disease subgroup. Those who suffered from more than one chronic disease within the subgroup were excluded from the comparison for that disease.

### Outcomes

PA was measured using Axivity AX3 wrist-worn triaxial accelerometers, collected from May 2013 until December 2015. Each individual had PA measured from their dominant wrist over a 7-day period at 100 Hz with a dynamic range of ±8 g with cut points set to 5-second epochs. Details on data processing can be found elsewhere.^7^

From the processed data, minutes of moderate and vigorous PA were calculated by considering the percentage of time spent above 100-milligravities (mg) activity intensity for moderate PA and above 400-mg intensity for vigorous PA.^12^ As both moderate and vigorous PA are derived from activity intensity and are more intuitive to comprehend, mean acceleration is not described in detail in this paper but can be found in Supplementary Table 1.

Of the 103 720 participants who provided accelerometry data, 7001 participants were excluded due to poor accelerometer wear time—defined as not having at least 3 days (72 hours) of data and or lacking data in each 1-hour period of the 24-hour cycle scattered over multiple days. A further 11 were excluded due to poor device calibration where recalibration by the preceding or subsequent measurement was not possible due to insufficient data and 2 were excluded due to discordant dates of device wear, leaving a total of 96 706 participants.

### Statistical analyses

Descriptive statistics are presented for anthropometric and lifestyle characteristics subdivided by chronic-disease status overall and sex in Table 1 . Additionally, geometric means [95% confidence intervals (CIs)] for activity intensity and minutes of moderate and vigorous PA adjusted for age and body mass index (BMI) are presented, subdivided by chronic-disease status and sex.

**Table 1.** Sample characteristics by presence or absence of chronic diseases and sex

**Characteristic**	**No chronic disease**		**Chronic disease**	
	Women (*n* = 31 901)	Men (*n* = 23 493)	Women (*n* = 22 547)	Men (*n* = 18 765)
Age (years)**^a^**	60.7 (7.7)	61.0 (8.0)	63.5 (7.5)	65.5 (7.1)
Weight (kg)**^a^**	69.0 (12.7)	84.4 (13.3)	71.7 (14.0)	85.7 (14.0)
Height (cm)**^a^**	163.6 (6.2)	176.8 (6.6)	163.1 (6.2)	176.0 (6.7)
BMI (kg/m^2^)**^a^**	25.8 (4.6)	27.0 (3.9)	26.9 (5.1)	27.7 (4.1)
Smoking status (%)				
Never	19 980 (62.6)	13 178 (56.1)	13 139 (58.3)	8781 (46.8)
Previous	9987 (31.3)	8364 (35.6)	7998 (35.5)	8336 (44.4)
Current	1864 (5.8)	1895 (8.1)	1342 (6.0)	1584 (8.4)
Alcohol intake (%)				
Daily or almost daily	6326 (19.8)	6500 (27.7)	3922 (17.4)	5337 (28.4)
Three or four times a week	8127 (25.5)	6982 (29.7)	4882 (21.7)	5128 (27.3)
Once or twice a week	8206 (25.7)	5859 (24.9)	5651 (25.1)	4530 (24.1)
One to three times a month	3916 (12.3)	1980 (8.4)	3053 (13.5)	1559 (8.3)
Special occasions only	3525 (11.1)	1235 (5.3)	3213 (14.3)	1208 (6.4)
Never	1779 (5.6)	920 (3.9)	1799 (8.0)	988 (5.3)
Highest qualification (%)				
None	1996 (6.3)	1480 (6.3)	2316 (10.3)	2211 (11.78)
GCSE or equivalent	5265 (16.5)	2516 (10.7)	4079 (18.1)	2329 (12.41)
A-level or equivalent	2285 (7.2)	1383 (5.9)	1340 (5.9)	950 (5.06)
Degree or equivalent	22 059 (69.2)	17 891 (76.2)	14 569 (64.6)	13 065 (69.62)
Ethnicity (%)				
White	30 729 (96.4)	22 672 (96.6)	21 811 (96.8)	18 173 (96.9)
Non-White	1162 (3.6)	808 (3.4)	724 (3.2)	583 (3.1)
Accelerometer wear time (hours/day)**^a^**	22.8 (2.3)	22.8 (2.3)	22.8 (2.2)	22.9 (2.22)
Accelerometer wear days/week**^a^**	6.6 (0.7)	6.7 (0.7)	6.7 (0.6)	6.7 (0.6)
Mean acceleration (mg/24 hours)**^b^**	28.4 (28.2–28.6)	27.5 (27.2–27.8)	26.3 (26.1–26.6)	25.1 (24.8–25.4)
Moderate activity (minutes/week)**^b^**	735.2 (726.6–743.9)	682.2 (672.5–692.0)	650.8 (640.3–661.4)	594.6 (583.6–605.9)
Vigorous activity (minutes/week)**^b^**	25.3 (24.7–25.9)	30.2 (29.3–31.1)	20.9 (20.3–21.5)	24.4 (23.6–25.2)

^a^Mean (standard deviation).

^b^Geometric mean adjusted for age and body mass index (95% confidence interval).

Adjusted geometric means (95% CI) for average acceleration and minutes of moderate and vigorous PA are presented for participants with a previously diagnosed chronic condition and those participants with no prior history of chronic disease adjusted for sex, age, BMI, smoking status, alcohol consumption, region, area deprivation using the Townsend deprivation index^13^ and ethnicity. Statistical comparison of means between disease-specific subgroups and those with no history of that disease were made using general linear models; e.g. mean minutes of moderate PA among participants with a previous diagnosis of any cancer were compared with participants with no previous history of a cancer diagnosis (Table 2). We conducted sensitivity analyses to determine the effect of ±25 mg change in PA on either side of activity cut-offs in individuals without chronic disease compared with individuals with cardiovascular disease.

Where statistical testing was used for comparison of the samples with an individual chronic disease to those without, we used a *p-*value of <0.001 to take into account the problem of multiple comparisons.

## Results

The analytic sample consisted of 96 706 participants aged between 43 and 79 years at the time they wore accelerometers. Overall, 44% of participants had one or more chronic diseases (Table 1). For within-disease category analyses, the following numbers were excluded due to the presence of two or more conditions within the category: 1 for infectious diseases; 593 for malignant cancer; 2728 for cardiovascular disease; 265 for endocrine and metabolic disorders; 70 for chronic neurologic disorders; 59 for mental health disorders; 15 for chronic haematological disorders; 375 for chronic respiratory disorders; 3396 for chronic gastrointestinal diseases; 684 for chronic genitourinary disorders; 14 for chronic ear, mastoid and hearing disorders; 603 for chronic eye disorders; and 697 for chronic musculoskeletal disorders. Compared with participants without chronic disease, both men and women with chronic disease were on average older, more likely to be ex-smokers, less educated and were less active, with lower activity intensity over 24 hours, lower moderate PA and vigorous PA levels per week. Compared with non-diseased individuals, those with chronic disease had lower alcohol intake.

**Figure 1.**
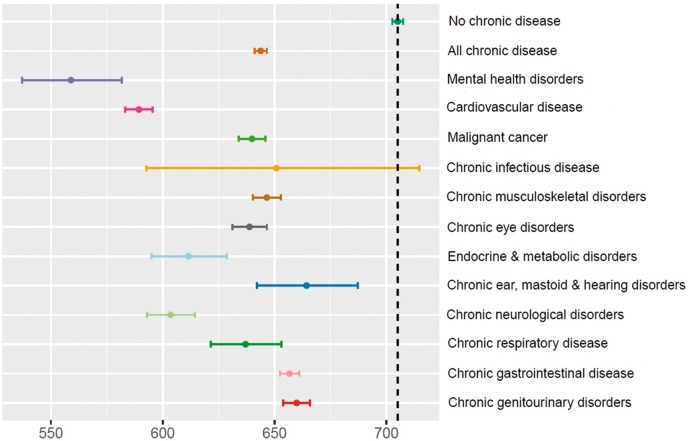
Geometric mean moderate activity in minutes per week for participants with and without chronic diseases.

Figure 1 presents moderate PA data for participants with and without chronic disease. Participants without chronic disease were found to undertake 61 minutes (9%) more moderate PA (95% CI: 57.6–65.0) per week than individuals with chronic disease, on average. Those with no chronic disease averaged 705 minutes per week of moderate PA whereas estimates for all chronic-disease subgroups were lower. The greatest difference in moderate PA was seen in individuals with mental health disorders whose mean was 559 minutes/week, followed by cardiovascular diseases at 589 minutes/week and chronic neurological disorders at 604 minutes/week. Furthermore, participants without chronic disease engaged in 3 minutes (11%) more vigorous PA (95% CI: 2.7–3.3) per week than individuals with chronic disease, on average (Supplementary Table 1, available as Supplementary data at *IJE* online). The greatest difference for vigorous PA was found in individuals with cardiovascular diseases with a mean of 23.1 minutes/week followed by chronic infectious diseases at 23.8 minutes/week and chronic neurological disorders at 23.9 minutes/week.

**Figure 2.**
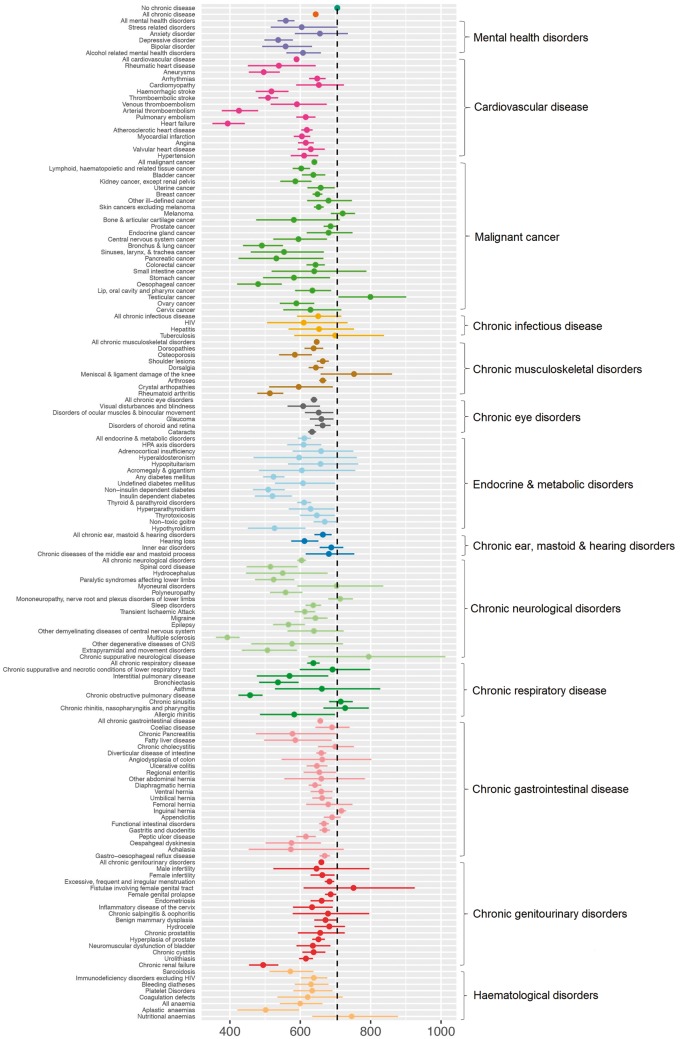
Geometric mean moderate activity in minutes per week for participants without chronic diseases and various chronic-disease subgroups.

The chronic-disease subgroups were populated by 147 individual disease diagnoses. Figure 2 provides moderate PA estimates and CIs for each. Almost half (67) were associated with a lower level of moderate PA when compared with individuals without chronic disease. Had we used the corrected Bonferroni *p*-value of <0.00034, we would have found only 55 of the individual disease diagnoses to be associated with moderate PA. For almost all chronic diseases, the point estimate of the mean moderate PA per week was lower than that for healthy individuals. The specific diseases with clearly lower weekly moderate PA include individuals with: systemic atrophies primarily affecting the central nervous system (70% lower), multiple sclerosis (44% lower), heart failure (44% lower), arterial thromboembolism (39.6% lower), COPD (35% lower), primary disorders of muscles (32% lower), oesophageal cancer (32% lower), bronchus and lung cancer (30% lower), chronic renal failure (30% lower) and aneurysms (30% lower).

Only 11 of the comparisons of vigorous-activity levels between chronic-disease subgroups and non-diseased had CIs that excluded a mean difference of zero, but a deficit in PA for diseased when compared with non-diseased was evident across diseases (Supplementary Table 1, available as Supplementary data at *IJE* online).

Current smokers without chronic disease were less moderately active than non-smokers without chronic disease (current smoker 643 minutes, previous 732 minutes, never 710 minutes) and also less vigorously active (current 23 minutes, previous 28 minutes, never 27 minutes) (data not shown). An inverse relationship was found between moderate PA and both age and BMI when narrower categories were used for stratification (Supplementary Figures 1 and 2, available as Supplementary data at *IJE* online).

The sensitivity analyses for ±25-mg change in moderate (100 mg) and vigorous (400 mg) cut-offs for acceleration changed the absolute minutes per week of activity, but the differences in PA between healthy participants and disease subgroups remained (Supplementary Table 2, available as Supplementary data at *IJE* online).

## Discussion

In this large prospective cohort study, we find clear evidence of lower PA levels among individuals with a prior diagnosis of chronic disease as compared with healthy individuals.

The greatest difference for moderate PA was seen for those with mental health disorders who averaged 2.4 hours less per week of moderate PA. This was followed by cardiovascular, neurological, endocrine and metabolic disorders, respiratory disorders, eye disorders and malignant cancer with lower levels of moderate PA between 1 and 2 hours per week. Of the 147 chronic diseases examined, accelerometry readings were lower for almost every chronic disease, with 67 being associated with lower moderate PA. This is despite the fact that the number of participants in each specific disease category was low for many and power-limited.

In a cross-sectional analysis of this kind, lower PA levels associated with disease might be explained by different mechanisms. The most readily anticipated is that the disease might be directly responsible for lower activity levels due to a reduced exercise capacity. This could be induced, e.g. by either reduced cardiopulmonary function or musculoskeletal dysfunction.^14^ However, not all of the diseases examined should have this effect; e.g. one might not expect gastrointestinal or genitourinary disorders to limit activity. As such, it is perhaps surprising that PA-measured objectively is lower across virtually all chronic diseases investigated. Some of the associations in these unexpected disease fields might be explained by the fact that physical inactivity leads to increased risk of these conditions and the individuals may have been habitually less active than their healthy counterparts for some time in order to develop the conditions. It would be surprising, however, if this, together with the disease effects described previously, accounted for the low PA found for all chronic diseases.

Possibly, just the fact of having a disease influences people to be less active, either through adoption of the sick role or concomitant undiagnosed depression. Regardless, the individuals so affected are deprived of the beneficial effects that PA might provide.^15^ An awareness of this and consequent implementations of a means to address it should be on the agenda of all clinicians and policymakers with a responsibility for patients with any chronic disease.

The mean moderate PA of 705 minutes per week for healthy individuals appears high in comparison to previous estimates based on self-report measures and the WHO guideline of 150 minutes minimum of leisure activity.^16^ However, this should not be surprising. Accelerometry captures habitual activity that might not necessarily be recalled and included in self-report and therefore might be expected to provide higher results. Matthews *et al.* recently reported that participants in the USA-based National Health and Nutrition Examination Survey (NHANES) study were estimated to take 1.7 hours of moderate to vigorous activity per day based on accelerometry measurement.^17^ This is not very different to the 1.67 hours per day we estimated here for the UK Biobank participants who were free of chronic disease.

### Limitations

The key comparison made here is between those for whom no evidence exists for a diagnosis of a chronic disease and those who are identified as having such a condition within hospital records. The accuracy of the conclusions drawn is therefore limited by the accuracy of coding within these data, which has been discussed elsewhere,^15^ as with all observational studies.

We have tried to control for the confounding effect of multi-morbidity by defining specific diagnoses as being present in the individual in the absence of other chronic diagnoses within that chronic-disease subgroup. The exclusion of diagnoses outside each chronic-disease subgroup for this purpose reduced sample sizes and limited examination of the research question for some diseases. The inclusion of those with only one disease also removed the possibility of examining the effects of multi-morbidity. Future work could address the implications of multi-morbidity on PA.

The response rate to invitations to become a participant in UK Biobank might affect the generalizability of the prevalence estimates we found.^18^ However, for this to produce the differences seen here between participants with and without chronic disease, there would have to have been a different association between having a disease and being active among respondents compared with non-respondents. This is possible but it seems much more likely that the inactive among those with chronic disease would have been the non-responders, the effect of which would be to bias the results towards the null.

The thresholds chosen for the Actigraph GT3x accelerometer for moderate and vigorous PA intensities at around 100 mg for three metabolic equivalents (moderate PA) and 400 mg for six metabolic equivalents (vigorous PA) were based on a small study of 30 Norwegian adults.^5^ Whilst this validation study was small, the authors conducted sensitivity analyses showing no appreciable change in the CIs if these thresholds were varied but did show modest variation in result magnitude. We also conducted sensitivity analysis on our data using different thresholds and found the main associations reported here remained.

## Conclusion

We found evidence of an association of physical inactivity with chronic disease that is broad. Clinicians and policymakers should be aware of the extent to which lower PA is associated with chronic disease and pay more attention to estimating PA in those with chronic disease, providing advice and programmes to address the problem. It is important to ensure that this subset of the population also accrues the health benefits obtained from adequate PA.

## Funding

This work was supported by the National Institute of Health Research (NIHR) Biomedical Research Centre, Oxford, and the British Heart Foundation Centre of Research Excellence at Oxford (grant number RE/13/30181 to A.D.). The sponsors played no role in study design, data collection, analysis and interpretation of data, in the writing of the report and in the decision to submit the paper for publication.

## Supplementary Material

dyy294_Supplementary_Data
