# Physical activity of UK adults with chronic disease: cross sectional analysis of accelerometer measured physical activity in 96 706 UK Biobank participants

**DOI:** 10.1093/ije/dyz148

**Published:** 2019-07-05

**Authors:** Joseph Barker, Karl Smith Byrne, Aiden Doherty, Charlie Foster, Kazem Rahimi, Rema Ramakrishnan, Mark Woodward, Terence Dwyer

First published online: 5 February 2019, *Int J Epidemiol*, doi: https://doi.org/10.1093/ije/dyy294

In the originally published version of this article an error appeared in the section Exposures, which began “Prevalence of chronic disease was identified using International Classification of Diseases, Ninth and Tenth Revisions (ICD 9 and 10) primary and secondary diagnoses from hospital admission data in England, Scotland and Wales and cancer registry data from 1997 up to accelerometer wear time for each individual (2010–13).”

However (2010-13) should have read (2013-2015). This has now been corrected.

