# Supplementary material for: Physical activity of UK adults with chronic disease: cross-sectional analysis of accelerometer-measured physical activity in 96 706 UK Biobank participants
Source: Int J Epidemiol. 2019 Feb 5;48(4):1167–74. doi: 10.1093/ije/dyy294 (PMC6693885; doi:10.1093/ije/dyy294)
Supplement: dyy294_Supplementary_Data [file dyy294_supplementary_data.zip › dyy294-Suppl_data/Supplementary_Data2.docx]

| **Supplementary Table 2**: Sensitivity analysis: Geometric mean moderate activity and vigorous activity (minutes per week) of participants with and without cardiovascular disease for +- 25mg either side of activity cut offs, adjusted for adjusted for age, sex, body mass index, smoking status, alcohol consumption, region, Townsend deprivation index, and ethnicity (95% CI). | | | | |
| --- | --- | --- | --- | --- |
| **Intensity cut off** | **Disease** | **Sample size** | **Geometric mean physical activity (minutes/week)** | **P-value** |
| **Moderate activity** |  |  |  |  |
| 75mg | No Chronic disease | 55,394 | 1,098.8 (1095.6-1101.9) |  |
|  | Any Cardiovascular disease | 7,040 | 956.5 (948.2-965.0) | <0.0001 |
| 100mg | No Chronic disease | 55,394 | 705.1 (702.7-707.5) |  |
|  | Any Cardiovascular disease | 7,040 | 589.4 (583.3-595.5) | <0.0001 |
| 125mg | No Chronic disease | 55,394 | 454.6 (452.9-456.4) |  |
|  | Any Cardiovascular disease | 7,040 | 367.4 (363.1-371.8) | <0.0001 |
| **Vigorous activity** |  |  |  |  |
| 375mg | No Chronic disease | 55,394 | 30.3 (30.1-30.6) |  |
|  | Any Cardiovascular disease | 7,040 | 25.4 (24.9-26.0) | <0.0001 |
| 400mg | No Chronic disease | 55,394 | 27.0 (26.8-27.2) |  |
|  | Any Cardiovascular disease | 7,040 | 23.1 (22.6-23.6) | <0.0001 |
| 425mg | No Chronic disease | 55,394 | 24.6 (24.5-24.8) |  |
|  | Any Cardiovascular disease | 7,040 | 21.3 (20.8-21.8) | <0.0001 |
