# Supplementary material for: Physical activity of UK adults with chronic disease: cross-sectional analysis of accelerometer-measured physical activity in 96 706 UK Biobank participants
Source: Int J Epidemiol. 2019 Feb 5;48(4):1167–74. doi: 10.1093/ije/dyy294 (PMC6693885; doi:10.1093/ije/dyy294)
Supplement: dyy294_Supplementary_Data [file dyy294_supplementary_data.zip › dyy294-Suppl_data/Supplementary_Data1.docx]

| **Supplementary Table 1**: Geometric mean activity (accelerometry readings in milligravities per 24hours), moderate activity and vigorous activity (minutes per week) of all participants, adjusted for adjusted for sex, age, body mass index, smoking status, alcohol consumption, region, Townsend deprivation index, and ethnicity (95% CI). | | | | | | | |
| --- | --- | --- | --- | --- | --- | --- | --- |
| **Disease** | **Sample size** | **Geometric mean activity (mg/day)** | **P-value** | **Geometric mean moderate activity (min/week)** | **P-value** | **Geometric mean vigorous activity (min/week)** | **P-value** |
| No chronic disease | 55394 | 27.9(27.8-27.9) |  | 705.1(702.7-707.5) |  | 27.0(26.8-27.2) |  |
| All chronic disease | 41312 | 26.4(26.3-26.4) | <0.001 | 643.8(641.0-646.6) | <0.0001 | 24.0(23.8-24.2) | <0.0001 |
| All chronic infectious disease | 73 | 26.7(25.1-28.3) | 0.15 | 650.8(592.7-714.7) | 0.09 | 23.8(19.4-29.3) | 0.23 |
| All malignant cancer | 7863 | 26.3(26.2-26.5) | <0.001 | 639.9(633.9-646.0) | <0.0001 | 24.4(23.9-24.9) | <0.0001 |
| All cardiovascular disease | 7040 | 25.2(25.1-25.4) | <0.001 | 589.4(583.3-595.5) | <0.0001 | 23.1(22.6-23.6) | <0.0001 |
| All endocrine & metabolic disorders | 854 | 25.8(25.3-26.2) | <0.001 | 611.6(594.9-628.7) | <0.0001 | 24.2(22.8-25.8) | 0.001 |
| All chronic neurological disorders | 2133 | 25.8(25.5-26.0) | <0.001 | 603.6(592.9-614.4) | <0.0001 | 23.9(22.9-24.9) | <0.0001 |
| All mental health disorders | 415 | 24.1(23.5-24.7) | <0.001 | 559.0(537.2-581.7) | <0.0001 | 23.4(21.3-25.7) | 0.003 |
| All chronic respiratory disease | 1066 | 26.5(26.0-26.9) | <0.001 | 637.1(621.5-653.1) | <0.0001 | 24.7(23.4-26.1) | 0.002 |
| All chronic gastrointestinal disease | 16809 | 26.7(26.6-26.8) | <0.001 | 656.8(652.4-661.1) | <0.0001 | 24.6(24.3-25.0) | <0.0001 |
| All chronic genitourinary disorders | 8267 | 26.8(26.7-27.0) | <0.001 | 659.9(653.9-665.9) | <0.0001 | 24.5(24.0-25.0) | <0.0001 |
| All chronic ear, mastoid & hearing disorders | 566 | 26.8(26.2-27.4) | <0.001 | 664.3(642.2-687.2) | 0.001 | 25.8(23.9-27.8) | 0.22 |
| All chronic eye disorders | 4837 | 26.4(26.2-26.6) | <0.001 | 638.8(631.1-646.6) | <0.0001 | 25.0(24.4-25.7) | <0.0001 |
| All chronic musculoskeletal disorders | 7759 | 26.7(26.5-26.8) | <0.001 | 646.6(640.3-652.8) | <0.0001 | 24.6(24.1-25.1) | <0.0001 |
| **INFECTIOUS** |  |  |  |  |  |  |  |
| Tuberculosis | 20 | 28.1(25.1-31.6) | 0.87 | 699.1(584.7-835.8) | 0.93 | 29.1(19.6-43.3) | 0.71 |
| Hepatitis | 33 | 26.5(24.3-29.0) | 0.27 | 653.3(568.4-750.9) | 0.28 | 21.8(16.0-29.8) | 0.18 |
| HIV | 19 | 25.8(23.0-29.1) | 0.21 | 609.7(507.5-732.4) | 0.12 | 22.5(15.1-33.3) | 0.36 |
| **MALIGNANT CANCER** |  |  |  |  |  |  |  |
| Cervix cancer | 39 | 26.0(24.0-28.3) | 0.11 | 629.0(553.4-714.9) | 0.081 | 24.3(18.2-32.4) | 0.46 |
| Ovary cancer | 100 | 25.9(24.6-27.3) | 0.005 | 588.8(543.5-637.9) | <0.0001 | 24.9(20.7-29.9) | 0.37 |
| Testicular cancer | 46 | 29.5(27.4-31.9) | 0.13 | 799.3(710.4-899.3) | 0.04 | 27.4(21.6-34.8) | 0.91 |
| Lip, oral cavity and pharynx cancer | 106 | 25.9(24.7-27.3) | 0.005 | 634.4(587.0-685.6) | 0.01 | 22.2(18.7-26.4) | 0.02 |
| Oesophageal cancer | 39 | 22.3(20.6-24.2) | <0.001 | 480.1(422.4-545.7) | <0.0001 | 18.6(13.6-25.5) | 0.02 |
| Stomach cancer | 25 | 25.5(23.0-28.2) | 0.08 | 581.9(496.0-682.8) | 0.02 | 22.8(15.8-32.7) | 0.35 |
| Small intestine cancer | 15 | 25.5(22.4-29.1) | 0.19 | 639.6(520.3-786.1) | 0.36 | 19.0(12.1-30.1) | 0.13 |
| Colorectal cancer | 453 | 26.4(25.8-27.0) | <0.001 | 643.2(619.4-668.0) | <0.0001 | 23.4(21.5-25.5) | 0.001 |
| Pancreatic cancer | 14 | 20.9(18.2-24.0) | <0.001 | 531.9(426.2-663.9) | 0.01 | 23.4(14.8-36.9) | 0.54 |
| Sinuses, larynx, & trachea cancer | 19 | 23.9(21.2-26.8) | 0.01 | 553.8(461.0-665.3) | 0.01 | 18.6(12.2-28.4) | 0.08 |
| Bronchus & lung cancer | 51 | 23.2(21.6-24.9) | <0.001 | 490.8(438.8-549.0) | <0.001 | 22.1(16.6-29.3) | 0.16 |
| Central nervous system cancer | 41 | 25.3(23.4-27.4) | 0.02 | 594.9(525.1-673.9) | 0.01 | 20.2(15.4-26.6) | 0.04 |
| Endocrine gland cancer | 74 | 26.9(25.4-28.6) | 0.26 | 680.4(620.1-746.6) | 0.45 | 24.4(20.1-29.8) | 0.32 |
| Prostate cancer | 946 | 27.2(26.7-27.7) | 0.01 | 686.0(668.0-704.4) | 0.07 | 25.9(24.5-27.4) | 0.15 |
| Bone & articular cartilage cancer | 16 | 24.9(21.9-28.3) | 0.08 | 581.7(476.3-710.3) | 0.06 | 22.1(13.7-35.6) | 0.41 |
| Ill-defined sites in the respiratory system and intrathoracic organs | 11 | 27.6(23.6-32.2) | 0.89 | 732.7(575.9-932.3) | 0.75 | 18.9(10.8-33.1) | 0.21 |
| Melanoma | 315 | 28.4(27.6-29.2) | 0.20 | 720.5(688.7-753.8) | 0.34 | 29.0(26.3-31.9) | 0.16 |
| Skin cancers excluding melanoma | 1935 | 26.7(26.4-27.0) | <0.001 | 652.4(640.5-664.6) | <0.001 | 25.6(24.6-26.6) | 0.01 |
| Other ill-defined cancer | 79 | 27.5(25.9-29.1) | 0.63 | 680.2(621.4-744.6) | 0.44 | 27.9(22.9-34.1) | 0.75 |
| Breast cancer | 1900 | 26.6(26.3-26.9) | <0.001 | 648.8(636.7-661.0) | <0.001 | 24.2(23.2-25.2) | <0.001 |
| Uterine cancer | 203 | 27.1(26.1-28.1) | 0.12 | 658.0(622.0-696.1) | 0.02 | 23.9(21.1-27.1) | 0.06 |
| Kidney cancer, except renal pelvis | 122 | 24.6(23.5-25.8) | <0.001 | 586.0(545.0-630.0) | <0.001 | 22.2(18.8-26.3) | 0.02 |
| Bladder cancer | 269 | 26.6(25.8-27.4) | 0.004 | 637.1(606.7-669.1) | <0.001 | 24.6(22.0-27.4) | 0.09 |
| Lymphoid, haematopoietic and related tissue cancer | 452 | 25.2(24.6-25.8) | <0.001 | 602.6(580.2-625.9) | <0.001 | 22.7(20.9-24.8) | <0.001 |
| **CARDIOVASCULAR** |  |  |  |  |  |  |  |
| Hypertension | 172 | 25.8(24.8-26.8) | <0.001 | 610.9(574.7-649.4) | <0.001 | 24.9(21.6-28.7) | 0.27 |
| Valvular heart disease | 189 | 26.3(25.3-27.3) | 0.002 | 629.9(594.2-667.8) | <0.001 | 25.2(22.0-28.8) | 0.30 |
| Angina | 579 | 26.1(25.5-26.6) | <0.001 | 615.7(595.4-636.7) | <0.001 | 24.0(22.2-25.9) | 0.003 |
| Myocardial infarction | 512 | 25.2(24.6-25.8) | <0.001 | 604.5(583.3-626.4) | <0.001 | 23.3(21.5-25.2) | <0.001 |
| Atherosclerotic heart disease | 1260 | 25.8(25.4-26.2) | <0.001 | 618.7(604.7-633.1) | <0.001 | 23.2(22.0-24.4) | <0.001 |
| Heart failure | 53 | 21.5(20.0-23.1) | <0.001 | 393.7(352.0-440.5) | <0.001 | 16.9(12.2-23.3) | 0.004 |
| Pulmonary embolism | 377 | 25.8(25.1-26.5) | <0.001 | 615.4(590.5-641.5) | <0.001 | 25.7(23.4-28.1) | 0.27 |
| Arterial thromboembolism | 47 | 23.1(21.4-24.9) | <0.001 | 425.6(378.7-478.4) | <0.001 | 27.9(20.7-37.6) | 0.84 |
| Venous thromboembolism | 37 | 25.4(23.4-27.7) | 0.03 | 590.4(517.7-673.3) | 0.008 | 23.9(17.4-32.8) | 0.44 |
| Thromboembolic stroke | 244 | 24.1(23.3-24.9) | <0.001 | 508.4(482.8-535.3) | <0.001 | 22.8(20.0-26.0) | 0.01 |
| Haemorrhagic stroke | 87 | 23.4(22.1-24.7) | <0.001 | 518.0(475.2-564.6) | <0.001 | 20.4(16.6-25.1) | 0.007 |
| Cardiomyopathy | 63 | 26.1(24.5-27.8) | 0.05 | 652.8(590.3-722.0) | 0.14 | 21.4(17.2-26.7) | 0.04 |
| Arrhythmias | 582 | 26.5(25.9-27.0) | <0.001 | 648.1(626.8-670.1) | <0.001 | 24.1(22.4-25.9) | 0.002 |
| Aneurysms | 89 | 23.2(22.0-24.5) | <0.001 | 496.0(455.6-539.9) | <0.001 | 20.2(16.4-25.0) | 0.007 |
| Rheumatic heart disease | 21 | 23.9(21.4-26.7) | 0.01 | 539.1(452.8-641.9) | 0.003 | 18.2(12.1-27.4) | 0.06 |
| **MENTAL HEALTH** |  |  |  |  |  |  |  |
| Alcohol related mental health disorders | 109 | 24.6(23.4-25.8) | <0.001 | 607.6(562.1-656.8) | <0.001 | 20.2(16.9-24.1) | 0.001 |
| Bipolar disorder | 43 | 24.5(22.7-26.5) | 0.002 | 558.2(493.4-631.5) | <0.001 | 25.4(18.9-34.0) | 0.67 |
| Depressive disorder | 125 | 23.6(22.5-24.7) | <0.001 | 537.2(499.9-577.2) | <0.001 | 23.5(19.7-28.0) | 0.121 |
| Anxiety disorder | 52 | 26.2(24.3-28.1) | 0.08 | 655.8(586.3-733.4) | 0.21 | 23.0(18.1-29.4) | 0.198 |
| Stress related disorders | 27 | 26.5(24.0-29.2) | 0.30 | 604.0(517.9-704.4) | 0.05 | 35.7(25.1-50.8) | 0.125 |
| **HAEMATOLOGICAL** |  |  |  |  |  |  |  |
| Nutritional anaemias | 25 | 28.6(25.9-31.7) | 0.60 | 746.1(635.7-875.7) | 0.49 | 31.7(22.9-43.8) | 0.34 |
| Aplastic anaemias | 22 | 23.0(20.6-25.7) | 0.001 | 501.6(423.0-594.8) | <0.001 | 21.3(14.4-31.7) | 0.24 |
| All anaemia | 67 | 25.4(23.8-27.0) | 0.003 | 599.7(543.9-661.4) | 0.001 | 25.5(20.5-31.9) | 0.61 |
| Coagulation defects | 31 | 26.7(24.3-29.3) | 0.35 | 621.1(536.7-718.7) | 0.09 | 24.7(17.9-34.0) | 0.58 |
| Platelet Disorders | 90 | 26.5(25.1-28.0) | 0.07 | 633.7(582.2-689.8) | 0.01 | 24.1(20.0-29.1) | 0.23 |
| Bleeding diatheses | 121 | 26.5(25.3-27.8) | 0.04 | 630.5(585.9-678.5) | 0.003 | 24.3(20.6-28.5) | 0.19 |
| Immunodeficiency disorders excluding HIV | 209 | 25.9(25.0-26.8) | <0.001 | 638.4(603.8-674.9) | <0.001 | 24.0(21.2-27.2) | 0.06 |
| Sarcoidosis | 58 | 24.7(23.1-26.4) | <0.001 | 571.8(514.4-635.7) | <0.001 | 23.3(18.3-29.7) | 0.23 |
| **ENDOCRINE & METABOLIC** |  |  |  |  |  |  |  |
| Hypothyroidism | 28 | 24.6(22.3-27.1) | 0.011 | 526.8(452.9-612.8) | <0.001 | 25.7(18.5-35.7) | 0.76 |
| Non-toxic goitre | 307 | 27.1(26.3-27.9) | 0.05 | 669.5(639.6-700.9) | 0.03 | 25.2(22.8-27.9) | 0.17 |
| Thyrotoxicosis | 118 | 26.5(25.2-27.7) | 0.03 | 647.0(601.1-696.5) | 0.02 | 27.4(23.3-32.2) | 0.88 |
| Hyperparathyroidism | 64 | 26.1(24.5-27.8) | 0.05 | 629.3(569.0-696.0) | 0.03 | 24.1(19.2-30.2) | 0.32 |
| Thyroid & parathyroid disorders | 741 | 25.9(25.4-26.3) | <0.001 | 610.8(592.9-629.1) | <0.001 | 24.4(22.8-26.1) | 0.003 |
| Insulin dependent diabetes | 68 | 24.0(22.6-25.5) | <0.001 | 520.9(472.7-573.9) | <0.001 | 21.8(17.3-27.5) | 0.07 |
| Non-insulin dependent diabetes | 89 | 23.8(22.6-25.2) | <0.001 | 508.9(467.3-554.3) | <0.001 | 21.1(16.9-26.3) | 0.03 |
| Undefined diabetes mellitus | 34 | 26.2(24.0-28.6) | 0.16 | 607.8(529.9-697.1) | 0.03 | 26.7(19.7-36.2) | 0.93 |
| Any diabetes mellitus | 217 | 24.1(23.3-24.9) | <0.001 | 523.8(495.9-553.2) | <0.001 | 21.4(18.7-24.4) | <0.001 |
| Acromegaly & gigantism | 13 | 25.0(21.7-28.8) | 0.14 | 604.5(484.3-754.4) | 0.17 | 19.5(11.5-33.0) | 0.22 |
| Hypopituitarism | 29 | 25.6(23.3-28.2) | 0.09 | 657.6(566.9-762.8) | 0.36 | 20.5(14.3-29.4) | 0.13 |
| Hyperaldosteronism | 11 | 24.2(20.7-28.2) | 0.07 | 596.5(468.8-759.0) | 0.17 | 22.4(12.8-39.2) | 0.51 |
| Adrenocortical insufficiency | 39 | 26.4(24.3-28.7) | 0.20 | 659.0(579.8-748.9) | 0.30 | 23.9(17.7-32.4) | 0.43 |
| HPA axis disorders | 110 | 25.3(24.1-26.6) | <0.001 | 609.5(564.8-657.8) | <0.001 | 22.2(18.5-26.6) | 0.03 |
| **NEUROLOGICAL** |  |  |  |  |  |  |  |
| Chronic suppurative neurological disease | 11 | 30.2(25.8-35.2) | 0.32 | 794.5(624.4-1010.9) | 0.33 | 30.7(18.6-50.6) | 0.62 |
| Systemic atrophies primarily affecting the central nervous system | 11 | 17.0(14.5-20.0) | <0.001 | 209.9(163.0-270.3) | <0.001 | 16.8(6.8-41.9) | 0.31 |
| Extrapyramidal and movement disorders | 29 | 24.5(22.2-27.0) | 0.008 | 506.6(435.5-589.2) | <0.001 | 24.4(17.0-35.0) | 0.58 |
| Other degenerative diseases of CNS | 13 | 25.6(22.2-29.5) | 0.24 | 576.7(462.1-719.8) | 0.08 | 28.3(16.2-49.4) | 0.88 |
| Multiple sclerosis | 104 | 21.4(20.3-22.5) | <0.001 | 392.7(362.1-425.8) | <0.001 | 20.8(16.7-25.8) | 0.02 |
| Other demyelinating diseases of central nervous system | 43 | 25.3(23.4-27.4) | 0.02 | 638.8(565.5-721.6) | 0.11 | 20.7(15.7-27.4) | 0.06 |
| Epilepsy | 112 | 23.5(22.3-24.7) | <0.001 | 566.3(524.3-611.6) | <0.001 | 20.6(17.2-24.7) | 0.003 |
| Migraine | 254 | 26.2(25.3-27.0) | <0.001 | 642.9(611.3-676.2) | <0.001 | 23.1(20.7-25.9) | 0.006 |
| Transient Ischaemic Attack | 314 | 25.7(25.0-26.4) | <0.001 | 612.5(585.4-640.9) | <0.001 | 24.7(22.3-27.4) | 0.10 |
| Sleep disorders | 634 | 26.9(26.3-27.4) | 0.001 | 636.8(616.6-657.7) | <0.001 | 24.7(23.0-26.6) | 0.02 |
| Mononeuropathy, nerve root and plexus disorders of lower limbs | 289 | 28.2(27.4-29.1) | 0.40 | 713.5(680.7-747.9) | 0.61 | 26.6(24.0-29.4) | 0.75 |
| Polyneuropathy | 103 | 25.1(23.8-26.4) | <0.001 | 558.2(515.7-604.2) | <0.001 | 24.8(20.6-29.9) | 0.37 |
| Myoneural disorders | 22 | 28.1(25.2-31.4) | 0.88 | 703.2(592.9-834.0) | 0.98 | 20.2(13.6-30.0) | 0.15 |
| Primary disorders of muscles | 11 | 22.5(19.2-26.5) | 0.01 | 477.4(370.8-614.6) | 0.002 | 28.0(14.7-53.3) | 0.92 |
| Paralytic syndromes affecting lower limbs | 61 | 23.8(22.3-25.4) | <0.001 | 524.2(472.8-581.3) | <0.001 | 20.3(15.6-26.4) | 0.03 |
| Hydrocephalus | 15 | 24.9(21.8-28.4) | 0.09 | 550.1(447.5-676.1) | 0.02 | 20.0(12.1-32.9) | 0.24 |
| Spinal cord disease | 37 | 23.5(21.6-25.7) | <0.001 | 515.0(449.0-590.7) | <0.001 | 16.2(11.5-22.9) | 0.004 |
| **RESPIRATORY** |  |  |  |  |  |  |  |
| Allergic rhinitis | 20 | 25.5(22.7-28.5) | 0.12 | 582.7(487.3-696.7) | 0.04 | 27.0(18.2-40.1) | 0.999 |
| Chronic rhinitis, nasopharyngitis and pharyngitis | 86 | 28.2(26.6-29.7) | 0.72 | 727.4(667.4-792.9) | 0.48 | 27.5(23.0-32.8) | 0.87 |
| Chronic sinusitis | 327 | 27.7(27.0-28.5) | 0.76 | 715.0(684.0-747.4) | 0.53 | 25.8(23.4-28.4) | 0.35 |
| Chronic obstructive pulmonary disease | 129 | 22.7(21.7-23.7) | <0.001 | 457.3(426.0-490.8) | <0.001 | 21.0(17.5-25.2) | 0.006 |
| Asthma | 13 | 26.6(23.0-30.6) | 0.50 | 661.3(529.8-825.4) | 0.57 | 24.1(14.6-39.8) | 0.65 |
| Bronchiectasis | 63 | 24.2(22.7-25.8) | <0.001 | 536.4(485.0-593.3) | <0.001 | 21.6(16.8-27.8) | 0.08 |
| Interstitial pulmonary disease | 21 | 24.0(21.5-26.8) | 0.01 | 569.4(478.2-677.9) | 0.02 | 19.2(13.0-28.5) | 0.09 |
| Chronic suppurative and necrotic conditions of lower respiratory tract | 32 | 28.3(25.8-30.9) | 0.77 | 691.7(600.6-796.6) | 0.79 | 31.5(23.4-42.5) | 0.32 |
| **GASTROINTESTINAL** |  |  |  |  |  |  |  |
| Gastro-oesophageal reflux disease | 1635 | 26.9(26.6-27.2) | <0.001 | 669.3(656.1-682.8) | <0.001 | 24.5(23.4-25.6) | <0.001 |
| Achalasia | 12 | 24.9(21.5-28.9) | 0.14 | 573.2(455.1-721.9) | 0.08 | 26.2(15.5-44.4) | 0.91 |
| Oespahgeal dyskinesia | 36 | 24.1(22.2-26.3) | 0.001 | 574.9(503.2-656.8) | 0.003 | 22.5(16.2-31.2) | 0.27 |
| Peptic ulcer disease | 360 | 25.8(25.2-26.6) | <0.001 | 615.8(590.3-642.4) | <0.001 | 24.5(22.2-27.0) | 0.05 |
| Gastritis and duodenitis | 1674 | 27.0(26.6-27.3) | <0.001 | 669.3(656.2-682.7) | <0.001 | 25.2(24.2-26.4) | 0.003 |
| Functional intestinal disorders | 1955 | 27.0(26.7-27.3) | <0.001 | 667.3(655.2-679.7) | <0.001 | 25.0(24.1-26.0) | <0.001 |
| Appendicitis | 612 | 27.6(27.0-28.1) | 0.30 | 690.5(668.5-713.2) | 0.21 | 27.2(25.4-29.1) | 0.90 |
| Inguinal hernia | 2641 | 28.3(28.0-28.6) | 0.001 | 716.7(705.2-728.3) | 0.02 | 27.2(26.3-28.1) | 0.71 |
| Femoral hernia | 72 | 28.2(26.6-30.0) | 0.68 | 679.3(618.2-746.5) | 0.44 | 29.9(24.2-37.0) | 0.35 |
| Umbilical hernia | 401 | 27.2(26.5-27.9) | 0.06 | 661.9(635.9-689.0) | 0.002 | 24.3(22.2-26.6) | 0.02 |
| Ventral hernia | 323 | 27.2(26.5-28.0) | 0.12 | 659.8(631.0-690.0) | 0.004 | 26.6(24.1-29.3) | 0.76 |
| Diaphragmatic hernia | 1007 | 26.3(25.9-26.8) | <0.001 | 641.8(625.7-658.3) | <0.001 | 24.0(22.7-25.4) | <0.001 |
| Other abdominal hernia | 22 | 25.9(23.2-28.8) | 0.18 | 659.8(556.4-782.3) | 0.45 | 20.0(14.2-28.3) | 0.09 |
| Regional enteritis | 138 | 26.5(25.4-27.7) | 0.03 | 654.3(611.1-700.5) | 0.03 | 23.9(20.6-27.8) | 0.11 |
| Ulcerative colitis | 343 | 26.5(25.8-27.3) | <0.001 | 647.1(619.7-675.7) | <0.001 | 24.3(22.1-26.7) | 0.03 |
| Angiodysplasia of colon | 19 | 27.0(24.0-30.5) | 0.63 | 662.6(548.7-800.1) | 0.52 | 41.9(26.5-66.0) | 0.06 |
| Diverticular disease of intestine | 1851 | 26.8(26.5-27.2) | <0.001 | 659.7(647.3-672.3) | <0.001 | 25.6(24.5-26.7) | 0.02 |
| Chronic cholecystitis | 130 | 27.1(25.9-28.4) | 0.23 | 699.6(652.0-750.6) | 0.83 | 25.1(21.5-29.3) | 0.34 |
| Fatty liver disease | 25 | 24.7(22.3-27.4) | 0.02 | 585.8(499.2-687.4) | 0.02 | 19.0(13.1-27.6) | 0.07 |
| Chronic Pancreatitis | 17 | 24.7(21.8-28.0) | 0.06 | 577.7(475.8-701.3) | 0.04 | 21.4(14.0-32.6) | 0.27 |
| Coeliac disease | 140 | 27.6(26.4-28.8) | 0.66 | 690.0(644.8-738.5) | 0.53 | 23.9(20.7-27.6) | 0.10 |
| **MUSCULOSKELETAL** |  |  |  |  |  |  |  |
| Rheumatoid arthritis | 139 | 23.5(22.5-24.6) | <0.001 | 513.6(479.6-550.0) | <0.001 | 20.9(17.6-24.8) | 0.003 |
| Crystal arthopathies | 29 | 25.9(23.5-28.4) | 0.12 | 595.5(513.3-690.8) | 0.03 | 22.9(16.6-31.7) | 0.32 |
| Arthroses | 4343 | 27.2(27.0-27.4) | <0.001 | 663.8(655.5-672.3) | <0.001 | 25.4(24.7-26.2) | 0.002 |
| Meniscal & ligament damage of the knee | 37 | 29.1(26.7-31.7) | 0.32 | 752.7(659.6-858.9) | 0.33 | 27.7(21.0-36.5) | 0.87 |
| Dorsalgia | 744 | 26.5(26.0-27.0) | <0.001 | 644.3(625.5-663.6) | <0.001 | 25.0(23.4-26.6) | 0.02 |
| Shoulder lesions | 1229 | 26.9(26.5-27.3) | <0.001 | 663.9(648.8-679.3) | <0.001 | 25.0(23.8-26.3) | 0.003 |
| Osteoporosis | 111 | 25.3(24.1-26.6) | <0.001 | 584.5(541.4-631.1) | <0.001 | 21.3(18.0-25.3) | 0.007 |
| Dorsopathies | 430 | 26.2(25.5-26.8) | <0.001 | 638.0(613.7-663.2) | <0.001 | 23.5(21.6-25.6) | 0.001 |
| **GENITOURINARY** |  |  |  |  |  |  |  |
| Chronic renal failure | 100 | 22.7(21.5-23.8) | <0.001 | 494.4(456.4-535.7) | <0.001 | 21.7(18.0-26.3) | 0.02 |
| Urolithiasis | 769 | 25.7(25.3-26.2) | <0.001 | 616.0(598.4-634.2) | <0.001 | 23.4(21.9-24.9) | <0.001 |
| Chronic cystitis | 275 | 27.1(26.2-27.9) | 0.06 | 637.8(607.8-669.4) | <0.001 | 26.1(23.5-29.0) | 0.53 |
| Neuromuscular dysfunction of bladder | 123 | 26.3(25.1-27.6) | 0.02 | 635.7(591.1-683.7) | 0.005 | 24.6(20.8-29.0) | 0.26 |
| Hyperplasia of prostate | 1116 | 26.6(26.2-27.0) | <0.001 | 651.7(635.8-667.9) | <0.001 | 24.7(23.4-26.0) | 0.001 |
| Chronic prostatitis | 66 | 26.2(24.6-27.9) | 0.06 | 656.6(595.0-724.5) | 0.16 | 28.1(22.7-34.8) | 0.72 |
| Hydrocele | 177 | 27.8(26.8-28.9) | 0.93 | 682.5(642.3-725.2) | 0.3 | 30.1(26.4-34.3) | 0.11 |
| Benign mammary dysplasia | 309 | 27.3(26.5-28.1) | 0.16 | 671.6(641.6-703.1) | 0.04 | 27.2(24.6-30.1) | 0.91 |
| Chronic salpingitis & oophoritis | 26 | 26.7(24.2-29.6) | 0.42 | 678.7(580.2-793.9) | 0.63 | 24.0(17.0-33.9) | 0.50 |
| Inflammatory disease of the cervix | 86 | 26.0(24.6-27.5) | 0.01 | 633.4(581.1-690.4) | 0.02 | 24.3(20.2-29.3) | 0.27 |
| Endometriosis | 307 | 26.4(25.7-27.2) | <0.001 | 660.7(631.1-691.7) | 0.005 | 24.1(21.9-26.6) | 0.02 |
| Female genital prolapse | 1559 | 27.6(27.3-28.0) | 0.28 | 686.2(672.1-700.5) | 0.02 | 24.8(23.6-26.0) | 0.001 |
| Fistulae involving female genital tract | 15 | 32.6(28.6-37.2) | 0.02 | 751.3(611.2-923.4) | 0.55 | 30.5(20.0-46.5) | 0.58 |
| Excessive, frequent and irregular menstruation | 2365 | 27.1(26.8-27.4) | <0.001 | 682.8(671.4-694.4) | <0.001 | 24.7(23.8-25.6) | <0.001 |
| Female infertility | 275 | 27.2(26.4-28.1) | 0.11 | 662.5(631.0-695.6) | 0.012 | 25.4(23.0-28.1) | 0.21 |
| Male infertility | 15 | 26.3(23.1-30.1) | 0.40 | 646.1(525.6-794.3) | 0.41 | 22.8(14.7-35.4) | 0.45 |
| **EAR** |  |  |  |  |  |  |  |
| Chronic diseases of the middle ear and mastoid process | 66 | 27.0(25.3-28.7) | 0.30 | 681.2(617.3-751.6) | 0.49 | 23.6(19.0-29.2) | 0.21 |
| Inner ear disorders | 303 | 27.3(26.5-28.1) | 0.18 | 688.0(657.1-720.4) | 0.31 | 27.8(25.2-30.7) | 0.58 |
| Hearing loss | 183 | 25.8(24.8-26.8) | <0.001 | 611.8(576.2-649.6) | <0.001 | 23.4(20.4-26.8) | 0.04 |
| **EYE** |  |  |  |  |  |  |  |
| Cataracts | 2979 | 26.4(26.1-26.7) | <0.001 | 633.3(623.7-643.1) | <0.001 | 24.9(24.1-25.8) | <0.001 |
| Disorders of choroid and retina | 673 | 26.7(26.2-27.3) | <0.001 | 663.5(643.3-684.4) | <0.001 | 26.0(24.4-27.9) | 0.29 |
| Glaucoma | 277 | 26.8(26.0-27.7) | 0.02 | 660.0(629.0-692.6) | 0.01 | 24.7(22.2-27.6) | 0.11 |
| Disorders of ocular muscles & binocular movement | 183 | 26.5(25.5-27.5) | 0.01 | 652.4(614.9-692.1) | 0.01 | 26.2(23.0-29.9) | 0.65 |
| Visual disturbances and blindness | 122 | 25.8(24.7-27.1) | 0.001 | 608.2(565.5-654.1) | <0.001 | 24.6(20.8-29.0) | 0.26 |
