# Supplementary figures and images for: Physical activity of UK adults with chronic disease: cross-sectional analysis of accelerometer-measured physical activity in 96 706 UK Biobank participants
Source: Int J Epidemiol. 2019 Feb 5;48(4):1167–74. doi: 10.1093/ije/dyy294 (PMC6693885; doi:10.1093/ije/dyy294)

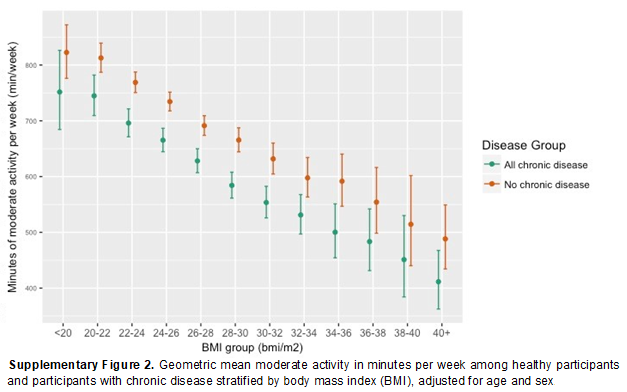

Supplement: dyy294_Supplementary_Data [file dyy294_supplementary_data.zip › dyy294-Suppl_data/Supplementary_Figure2.tif]

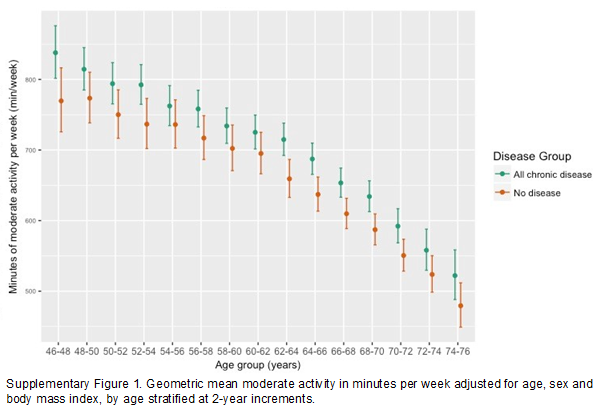

Supplement: dyy294_Supplementary_Data [file dyy294_supplementary_data.zip › dyy294-Suppl_data/Supplementary_Figure1.tif]
